# Supplementary material for: Patterns of Intron Gain and Loss in Fungi
Source: PLoS Biol. 2004 Nov 30;2(12):e422. doi: 10.1371/journal.pbio.0020422 (PMC532390; doi:10.1371/journal.pbio.0020422)
Supplement: Table S1 — Also available at http://genes.mit.edu/NielsenEtAl/. (4.3 MB ZIP). [file pbio.0020422.st001.zip › NielsenEtAl/html/218.html]

AN9007.1.NCU06895.1.MG03375.1.FG06068.1


```
 CLUSTAL W (1.82) Multiple Sequence Alignments - Introns Inserted


Sequence 1: NCU06895.1	539 aa
Sequence 2: MG03375.1	504 aa
Sequence 3: FG06068.1	531 aa
Sequence 4: AN9007.1	523 aa
Alignment Length: 545 aa
Number Identitical Residues: 176 aa
Alignment Score (without introns) 10763


MG03375.1 	----MENLCTWLGLLAGAVFLY0DHSWLGYQMSTS--FTMPGEETAILNFG-----ACTK
NCU06895.1	MVFGAAHLQLWLGALVVLGAVY0TSCLIFYRLFLHPLAKYPGPFLAKLTDGYMAYHAFKG
FG06068.1 	MTVDLAHLWVYLGLLVGGGLFY0GFSIVIYRVFFHPLAKYPGPFMAKITDAYQLYHAWKG
AN9007.1  	-MLQLLLPRSWPAALLAAGTIL0SFVWIAYYRFIHPLSRVPGPFLASVTPLVQLYHGLKG
          	          : . *     .      : *     .    **   * :.       . . 

MG03375.1 	N-------------T0VVRFGPNCISVNSATALKDVYGFRANVRKAEFYDAFVHP--APN
NCU06895.1	DRHLEFWRMHEKYGK~FVRFGPNSLSVNSNTALKDIYGFKANVRKAEFYDAFVHP--APN
FG06068.1 	DRQIEFWRMHEKYGP~VVRFGPNLVCFNSNKALKDIYGFRSNVRKAEFYNAFVHP--TAN
AN9007.1  	DRHLWIYELHQRYGD~HVRLAPNFVSINNVEGLHKIYGHGNKFRKADFYNGFLAIPGVYN
          	:         .  .   **:.** :..*.  .*:.:**.  :.***:**:.*:  ... *

MG03375.1 	THNARDRDLHARKRRVLSHAFSDNAIKQMERYILANERTFCAEIGRGASP------DSKG
NCU06895.1	THNARDRDLHARKRRVLAHAFSDSAIKEVERYILSNIRTFCDAIGDLGRPSFAPLADKKG
FG06068.1 	THNTSDKEVHARKRRVMSQAFSESAMKEMQRYILGNVRTFCEQIGMLEGSG----EDNKG
AN9007.1  	THNAIDKLVHGRKRRVLSQAFSDTALKGMEDVMLLHVRQLCSILGRERPTS----QSGDK
          	***: *: :*.*****:::***:.*:* ::  :* : * :*  :*    ..     . . 

MG03375.1 	WSTPKNMSDWCNYLAMDILGDLAFGKAFHMLESEDNRYALDMVGAAAQRHLI0CGTMPII
NCU06895.1	WSSPKNMSDWCSWLAMDILGDLCFGKAFHMLERPDNRYAVDLVSVAAHRHLI~CGTMPML
FG06068.1 	WTKPRKMSDWCNYLAMDILGDLCFGKAFHMLESPTNRFALQLVEAATTRHLL0CGTMPIV
AN9007.1  	DGATFNMANWFGYLTYDVMGELCFGKSFDMLIDGAKRRMIHLVDRAAYRHYV0CGLWMPL
          	   . :*::* .:*: *::*:*.***:*.**    :*  :.:*  *: ** : **    :

MG03375.1 	DKLSLDKVLFPKIAAGRAKYMKYSRA----QLTERTKLGDETDRRDFFYHLLRARDPETG
NCU06895.1	DKLSLDRIFLRKIASGRAQYMAYSRQ----QLAERTALGDETDRRDFFYYLLKARDPETG
FG06068.1 	NKLNLDKILFPGLAAGRARYMAYSKG----QLTERTKLGEETDRRDFFYYLLKARDPETG
AN9007.1  	HRWHLDQIFIRRLTNDRWNFIMESRQEANIRAKERTSLGQDA-KKDFFYYLLNARDPETG
          	.:  **::::  :: .* .::  *: .:. :  *** **::: ::****:**.*******

MG03375.1 	QGFSTPELWGESNLL2IIAGSDTTSTAMAATLFYLCRNPAALARVTEEVRTAFSSVEDIK
NCU06895.1	QGFTTPELWGESNLL~IIAGSDTTSTAMAATLFYLVRNPTALARVTAEIRSRFASVEGIH
FG06068.1 	QGFSTPELWSESNLL2IIAGSDTTSTAMAATLFYLVRCPRALKRVTEEIRSKFNDVEEIC
AN9007.1  	KGLATQELWGEANVL~MIAGSDTTSTSLSAAIFYLVRNPHALEKLKNEVRSHFSDVEEIV
          	:*::* ***.*:*:* :*********:::*::*** * * ** ::. *:*: * .** * 

MG03375.1 	QGPQLNGLTYLRACIDEAMRLSPSVGGILPREVLAGGITVDGETLPEGTVVGTPHYAIHH
NCU06895.1	QGPSLQSCTYLRACIDEAMRLSPSVGGLLPREVLPGGITIDGEHVPQGTIIGVPHYTIHH
FG06068.1 	QGATLASCTYLRACIDEAMRMNPSVGGILPREVLSGGMTIEGHVIPEGTVVGVPHYTIHH
AN9007.1  	TGSKLNQLTYLKACIDEAMRLAPAVPGSIPREASDPVVTVDGLVLPEGTGCGTPPYCIHR
          	 *. *   ***:********: *:* * :***.    :*::*  :*:**  *.* * **:

MG03375.1 	NEDYYPDPFAYAPERWIAG---SVLKSGRTVG-EADIQAAQSAFCPFSVGPRGCIGKGLA
NCU06895.1	NAAYYPDPWSYTPERWLSTRSPSEKTSGAAMQTEEQIQLAQSAFCPFSIGPRGCIGKGLA
FG06068.1 	NESYYPSPYEYVPERWLVG---TLNPLTGQNTTEAEVALASSAYCPFSVGPRGCIGKGLA
AN9007.1  	RPDYYREPLSYLPDRWIEG-STCKTADAAWTVTREEVDLARKAFCPFSIGPRGCIGKSMA
          	.  ** .*  * *:**:   :.          :. ::  * .*:****:********.:*

MG03375.1 	YVEMSTTLARTLFTYDMRKAVGVEDPSEGGKPGAEWGRHRESEFQLVDTFTSLKEGPICE
NCU06895.1	YIEMSITLARVLFLYDLRRAVGVDDPAEGGKKGAEYGRHRVGEMQLVDTFTSTKDGVMVE
FG06068.1 	YVEMTNTLARTMYMYDMRKAVGIVDPAEG-NPKNEWGRHRPGEMQLVDIFTSAKNGPMVE
AN9007.1  	LMEMRVTLARLMYLFDFELADAT-------------GEDENGHFKMVDHFVVSKTGPNVI
          	 :**  **** :: :*:. * .              *... ..:::** *.  * *    

MG03375.1 	~FRER-DLSA
NCU06895.1	~FRPREDLAA
FG06068.1 	~FRKADHMSS
AN9007.1  	0NIITRSL--
          	       :
```
